# Air Pollution Within the Public Health Exposome Framework: Impacts on Cardiovascular Health

**DOI:** 10.1007/s40471-026-00391-z

**Published:** 2026-04-27

**Authors:** Lucia D. Juarez, Andrzej Kulczycki, Gabriela R. Oates, Kai Zhang, Gokhan M. Mutlu, Sairam Parthasarathy

**Affiliations:** 1 General Internal Medicine and Population Science, Heersink School of Medicine, University of Alabama at Birmingham, BDB D820B, 1808 7th Ave S, Birmingham, AL USA; 2 Department of Health Policy and Organization, School of Public Health, University of Alabama at Birmingham, Birmingham, AL USA; 3 Pulmonary, Allergy and Critical Care Medicine, Heersink School of Medicine, University of Alabama at Birmingham, Birmingham, USA; 4 Department of Population and Community Health, The University of North Texas Health Science Center at Fort Worth, Fort Worth, TX USA; 5 Section of Pulmonary and Critical Care Medicine, University of Chicago, Chicago, IL USA; 6 University of Arizona, Tucson, AZ USA

**Keywords:** Air pollution, Cardiovascular disease, Exposome, Lifecourse epidemiology, Environmental health, Social determinants

## Abstract

**Purpose of Review:**

Ambient air pollution drives cardiovascular disease (CVD), yet single-pollutant models overlook how risk emerges within broader environmental and structural contexts. This review applies the Public Health Exposome (PHE) framework, which integrates natural, built, social, and policy environments, to show how multilevel systems shape CVD vulnerability.

**Recent Findings:**

Extreme heat, humidity, and climate variability modify pollutant toxicity, while structural inequities, discriminatory policies, and inadequate planning intensify exposures in marginalized communities. Cumulative psychosocial stress further amplifies inflammatory responses, activating the endo-exposome. Multi-omics studies reveal that pollution alters epigenomic, transcriptomic, and metabolomic pathways linked to inflammation, oxidative stress, and endothelial dysfunction. Emerging tools like machine learning, high-resolution exposure modeling, graph-based analytics, and wearable sensing, enable integration of environmental data and support systems-level approaches.

**Summary:**

The PHE framework illustrates how layered environmental and structural stressors accumulate to elevate CVD risk. Reducing this burden requires coupling PHE science with equitable public policy.

## Introduction

A recent Global Burden of Disease study [1] shows that cardiovascular disease (CVD) remains the leading global cause of death, accounting for nearly half of all non-communicable disease mortality. Growing evidence demonstrates that environmental exposures, particularly long-term exposure to ambient air pollution, play a significant role in this burden [2]. Recent global estimates attribute nine million deaths annually to poor air quality, underscoring air pollution as a leading environmental determinant of premature mortality [2] with profound socio-economic impacts [3]. Air pollution has become increasingly studied as an environmental risk factor for CVD, yet it represents only one component of the broader, interconnected exposome.

The exposome encompasses the totality of environmental influences, from conception through late life [4–6]. Air pollution operates within a broader network of chemical, physical, social, behavioral, and policy-related exposures that collectively influence CVD risk, intersect across biological pathways, and generate cumulative and synergistic effects overlooked in single-pollutant models. However, existing CVD epidemiology has largely examined fine particulate matter (particles ≤ 2.5 microns in diameter, PM₂.₅), nitrogen dioxide (NO₂), ozone (O_3_), and other pollutants separately, with limited integration of the natural, built, and social context in which they occur. Key gaps persist in understanding how mixtures of pollutants co-occur with heat, noise, psychosocial stress, and neighborhood disadvantage; how structural inequities concentrate multiple hazards; and how biological processes, such as inflammation, oxidative stress, and epigenetic dysregulation, encode the impact of these exposures over time.

**Fig. 1:**
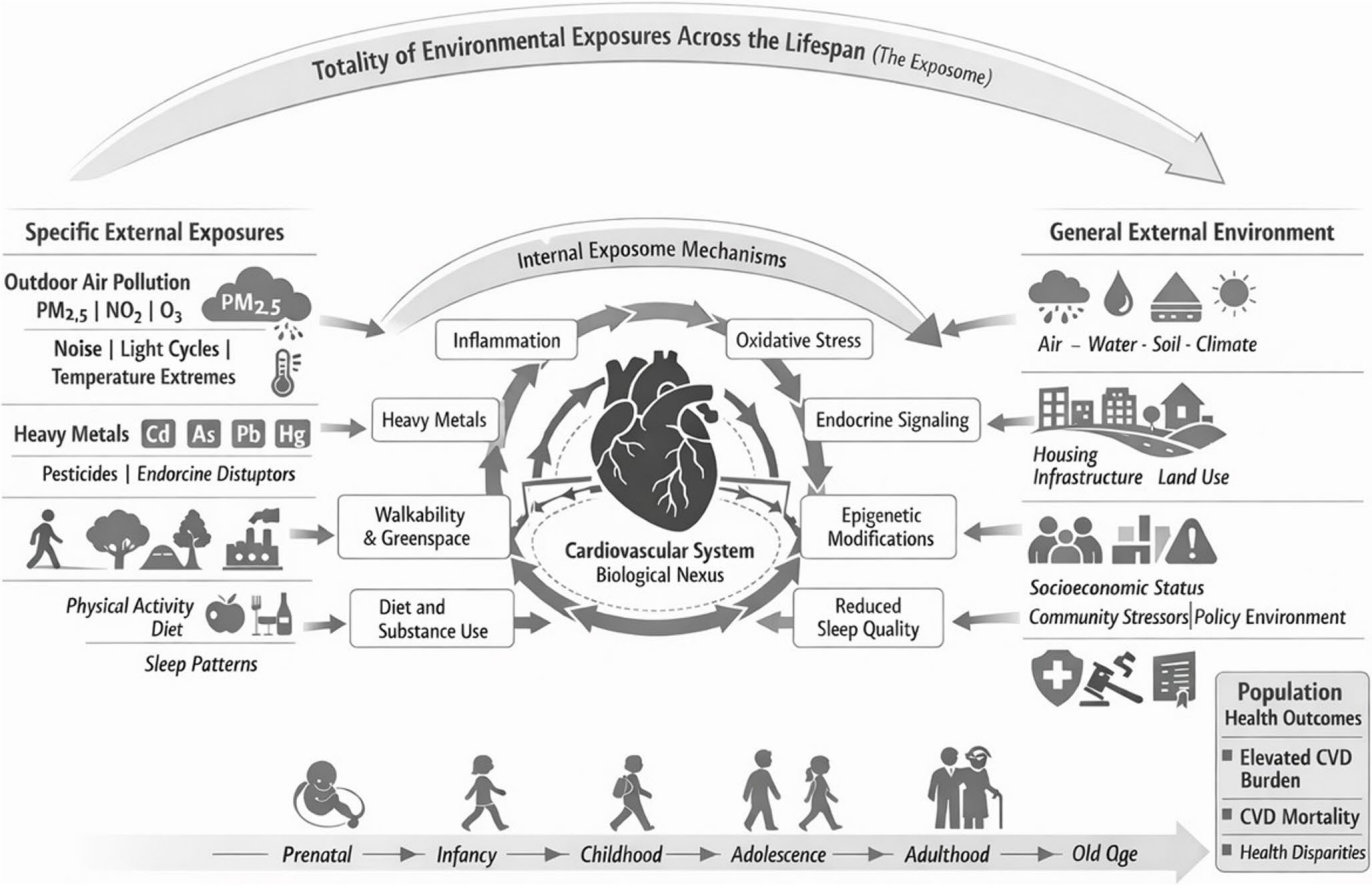
The Public Health Exposome Framework for Cardiovascular Disease Risk

In 2026, the American Heart Association (AHA), American College of Cardiology (ACC), European Society of Cardiology (ESC), and World Heart Federation (WHF) issued a joint statement urging the field to address air pollution and related environmental stressors as urgent, preventable determinants of CVD [7]. Responding to this call, this review situates long-term air pollution exposure within the Public Health Exposome (PHE) framework [5] to synthesize evidence on CVD impacts and identify scientific, methodological, and policy gaps. The PHE framework organizes external exposures across the natural, built, social, and policy environments and links them to biological responses through the endo-exposome (Fig. 1). Grounding the review in this systems-oriented perspective allows for a more integrated evaluation of how complex, multilevel exposures contribute to CVD risk and where opportunities exist for prevention and policy action.

## The Public Health Exposome Framework

The Public Health Exposome (PHE) framework places air pollution as one component within a multidimensional system of natural, built, social, and policy environments that collectively shape CVD risk across the lifecourse [4, 5, 8, 9]. This perspective moves beyond pollutant-specific analyses by emphasizing how exposures cluster and interact. Ambient pollution co-occurs with extreme temperatures, wildfire smoke, humidity, and traffic emissions, producing cumulative and synergistic cardiometabolic effects that are not captured when exposures are evaluated independently [10].

The built environment further structures exposure patterns and resilience. Walkability, green space, transit access, and housing quality influence both pollution levels and opportunities for health-promoting behaviors, whereas proximity to major roadways, and substandard housing increase exposure to PM₂.₅, NO₂, and indoor combustion pollutants [11, 12]. These features intersect with longstanding socioeconomic inequities and discriminatory land-use practices that concentrate multiple hazards in historically marginalized communities. Such structural forces heighten extrinsic vulnerability and amplify biological susceptibility through chronic stress, heightened inflammation, and reduced adaptive capacity [13, 14].

The PHE framework also incorporates temporal dimensions, highlighting critical windows, from prenatal development through older adulthood, when environmental exposures may exert durable CVD effects [15]. By integrating the endo-exposome, the framework links external exposures to internal biological responses, supported by multi-omics tools [16] capable of characterizing pathways related to oxidative stress, immune activation, endothelial dysfunction, and epigenetic regulation. Finally, advances in measurement and analytics, including satellite-derived data, low-cost sensing, exposure network modeling, and machine-learning approaches, enhance the precision with which this complex environmental system can be characterized [17]. Applied within the PHE framework, these tools enable a more holistic understanding of cumulative environmental burden and help identify leverage points for targeted prevention and policy interventions.

### Air Pollution

Air pollution arises from a mix of natural and human-made sources that emit primary pollutants such as PM, sulfur dioxide (SO_2_), and nitrogen oxides (N_x_O_x_), and generate secondary pollutants like O_3_, NO₂ and sulfates (SO_4_) through atmospheric reactions [18–20]. Extensive evidence shows that even low concentrations of PM₂.₅ contribute to premature mortality and CVD disease [21]. PM varies by size (PM_10_, PM₂.₅, ultrafine particles), with smaller particles penetrating deeper into the respiratory and vascular systems and driving systemic inflammation [13] and oxidative effects [22]. Toxicity is influenced by particle composition, size, oxidative potential, and meteorological conditions [20, 23]. In 2024, the U.S. Environmental Protection Agency (EPA) finalized new National Ambient Air Quality Standards (NAAQS) for fine particle pollution (soot), lowering the annual standard to 9 µg/m³ to improve public health, and set new standards for power plant emissions [24]. However, these moves were reversed one year later [25].

Anthropogenic sources, such as traffic emissions, industrial activities, and fossil fuel combustion, remain dominant contributors, while natural sources such as dust storms, wildfires, and sea spray increasingly shape regional air quality. Wildfire-related PM₂.₅ is also harmful due to its high oxidative potential and long-range transport [26]. Traffic emissions generate ultrafine particles and reactive gases including NOx, while sulfur oxides arise from combustion of sulfur-rich fuels. Atmospheric chemistry involving NOx, volatile organic compounds, and humidity drives formation of secondary sulfates and nitrates, elevating PM₂.₅ even in areas distant from emission sources [27, 28].

Exposure is further modified by features of the built and social environment. Poor housing quality, limited ventilation, and proximity to major roadways increase pollutant infiltration [29], while socioeconomic disadvantage [30] and discriminatory land-use patterns [31] concentrate exposures in historically marginalized communities. These multilevel influences underscore that air pollution is best understood as part of the broader exposome, interacting with co-occurring environmental and social stressors that shape CVD risk across the lifecourse.

As summarized in Table 1, these dynamics reflect a multidimensional exposure milieu in which chemical pollutants intersect with physical conditions, such as heat, humidity, and noise, as well as built environment factors, social stressors, and structural policy contexts. Together, these co-occurring exposures create interaction pathways, ranging from oxidative stress to autonomic and neuroendocrine disruption, that intensify vulnerability to ambient air pollution and contribute to persistent CVD disparities.

**Table 1:** Exposure Mixtures Matrix: Multidimensional Interactions With Ambient Air Pollution

Exposure Domain	Representative Exposures	Mechanistic Interaction With Air Pollution
Chemical	PM₂.₅, NO₂, O₃, volatile organic compounds (VOCs)	Potentiates oxidative stress, promotes systemic inflammation, and exacerbates cardiopulmonary toxicity
Physical	Ambient heat, humidity, environmental noise	Heat amplifies pollutant toxicity via thermoregulatory strain; noise augments autonomic nervous system activation and stress-related susceptibility
Built Environment	Traffic density, substandard housing, indoor particulate sources	Increases cumulative exposure burden and alters time–activity patterns, intensifying chronic inhalation and contact pathways
Social Stressors	Structural discrimination, socioeconomic deprivation, community violence	Heightens allostatic load, dysregulates neuroendocrine stress pathways, and amplifies inflammatory responses to pollutants
Policy and Structural Factors	Zoning practices, regulatory enforcement gaps, land-use policies	Spatially concentrates multi-hazard exposures and perpetuates environmental inequities in marginalized communities

### Epidemiologic Evidence Linking Long-Term Air Pollution Exposure with CVD

Well-established epidemiological evidence demonstrates that long-term exposure to air pollution, particularly PM₂.₅, increases the risk for atherosclerotic ischemic heart disease, myocardial infarction, stroke, heart failure, hypertension, arrhythmia and dyslipidemia [32–37]. Meta-analyses consistently report that chronic PM₂.₅ exposure increases CVD mortality by approximately 5–10% per 10 µg/m³ increase in PM₂.₅ [34], with associations evident even at concentrations below current regulatory limits.

Long-term exposure to PM_2.5_ contributes to ischemic heart disease [38], including higher incidence of myocardial infarction [34] and accelerated atherosclerotic plaque development [39]. Subclinical markers such as coronary artery calcium [40], carotid intima-media thickness [41], endothelial dysfunction [42] and arterial stiffness [43] provide strong mechanistic support for these associations. In addition, evidence for cerebrovascular outcomes shows that chronic PM₂.₅ exposure increases risks of stroke incidence, stroke mortality and cerebral small vessel disease [44, 45], with heightened vulnerability among older adults [46] and individuals with metabolic conditions [36, 47].

Air pollution also plays a role in heart failure, with studies linking long-term exposure to increased incidence [48] and hospitalizations [49], likely through pathways involving systemic inflammation [50], autonomic imbalance [51], and ventricular remodeling [52]. Furthermore, long-term exposure contributes to cardiometabolic precursors of CVD, hypertension [33, 36], dyslipidemia [53], insulin resistance [54], obesity [55], and type 2 diabetes [55], highlighting early pathways through which pollution increases downstream CVD risk.

Although evidence for nitrogen dioxide (NO₂) and other co-pollutants varies, the overall body of research demonstrates that chronic air pollution exposure is a pervasive, multisystem contributor to CVD across the life course [56, 57].

### Biological Mechanisms: Oxidative Stress, Inflammation, and Endothelial Injury

Environmental stressors across the exposome, including air pollutants, chemical toxicants, radiation, heat, and socioeconomic adversity, activate a cascade of biological pathways that converge on oxidative stress, inflammation, and endothelial dysfunction [58]. These exposures initiate neuroendocrine stress responses that elevate catecholamines and cortisol, contributing to allostatic load (AL), a multisystem marker of cumulative physiological strain. AL, also known as the “wear and tear” on the body over a lifetime, is an objective measure that quantifies the level of stress experienced throughout one’s life.

Air pollution is a particularly potent driver: PM₂.₅, NO₂, and ozone exposures have been linked to circadian rhythm disruption, dysregulation of stress hormones, and altered expression of circadian and metabolic genes [59]. Experimental evidence demonstrates that PM₂.₅ induces insulin resistance, reduced energy expenditure, and disrupted thermogenesis, effects that reverse when exposure ceases, while human studies show strong correlations between PM₂.₅ and components of the circadian-metabolic syndrome, including sleep disturbance and depression [59]. As a result, chronic environmental stress increases CVD vulnerability by impairing homeostatic balance and amplifying oxidative and inflammatory signaling.

These biological disturbances interact with chemical and physical pollutants such as lead, tobacco smoke [55], and ionizing radiation, which independently promote oxidative stress, immune activation, and endothelial damage. Long- and short-term epidemiologic studies, supported by imaging and experimental research, demonstrate that particulate matter promotes endothelial dysfunction, vascular stiffness, subclinical atherosclerosis, and development of vulnerable plaques through oxidative, inflammatory, thrombotic, and vasoconstrictive pathways. Cohort studies such as Heinz Nixdorf Recall, KORA, MESA and CARDIA [40, 41, 60] consistently link higher PM₂.₅ and traffic-related pollutants with increased carotid intima–media thickness, arterial calcification, arterial inflammation, and major CVD events. Together, these findings underscore oxidative stress, inflammation, and endothelial injury as central, interconnected biological mechanisms through which the exposome exerts its CVD effects.

## The Natural Environment

### Heat and Humidity

Heat waves are associated with increased CVD mortality. Extreme heat activates sympathetic responses, dehydration-related electrolyte imbalances, and systemic inflammation, increasing the incidence of hypertension and acute myocardial infarction, particularly among those with existing disease [61, 62]. These physiologic stresses occur alongside shifts in ambient air pollution, a dominant natural exposome stressor [3]. Temperature-sensitive ozone (O₃) accelerates airway inflammation, oxidative stress, endothelial dysfunction, and atherosclerotic progression, with heat intensifying its photochemical formation [42]. Wildfire smoke, drought conditions, and acrolein-rich traffic emissions further compound pollutant toxicity and population exposure [26, 27].

Humidity adds an additional layer of CVD stress by modifying both thermoregulation and atmospheric chemistry [28]. Extremes of high and low relative humidity impair heat dissipation and elevate CVD mortality, while long-term exposure to higher summer specific humidity and its variability is associated with increased CVD hospitalization [49]. Humidity also influences O₃ dynamics, affecting radical formation and pollutant persistence in urban air masses [61]. Methodologic evidence from multi-city analyses indicates that apparent temperature is the strongest predictor of heat-related all-cause mortality, whereas absolute humidity frequently emerges as a top predictor across cause-specific mortality outcomes [62]. These metrics remain central to accurately characterizing heat–health relationships in a warming climate.

### The Built Environment

The built environment, including transportation infrastructure, land-use patterns, green space distribution, and the local food environment, shapes CVD health through dynamic interactions with pollution exposures [63, 64]. Walkable, mixed-use neighborhoods encourage physical activity and reduce car dependence [11, 13, 63], yet residents often face higher exposure to traffic-related air pollution and noise, both of which drive oxidative stress, autonomic dysregulation, and endothelial injury [11]. Conversely, car-dependent areas may lower pollution exposure but contribute to sedentary behavior and social isolation [12, 64]. Food environments compound these spatial risks: neighborhoods with poor walkability and low greenness frequently lack supermarkets and affordable fresh foods, increasing food insecurity and reliance on calorie-dense ultra-processed foods [11]. These dietary constraints heighten inflammation, dyslipidemia, and insulin resistance, amplifying the biological effects of pollution. Evidence from an umbrella review [11] highlights strong, consistent relationships between air pollution, noise, and CVD, with the greatest burden borne by socially vulnerable communities where adverse built features, food insecurity, and environmental exposures co-occur. Understanding these interlocking pathways is essential for designing interventions that jointly address pollution, mobility, and nutrition.

### Urban Green Spaces and Pollution Interactions

Urban green spaces, parks, street trees, community gardens, and biodiverse natural areas, modify CVD risk by simultaneously influencing physical activity, pollution exposure, thermal stress, and food access [12]. Green vegetation can buffer particulate matter, lower ambient temperatures, and reduce noise [63], thereby mitigating pollution-related endothelial dysfunction and autonomic stress. However, these benefits depend on quality, biodiversity, and spatial placement [65]: heavily trafficked parks or green corridors adjacent to major roadways may expose users to elevated PM₂.₅ and NO₂, partly offsetting CVD gains. Studies from other countries consistently show lower CVD mortality in greener areas [11, 12]. However, analyses from vulnerable populations using CARDIA data reveal complexities [66], such as greater greenness lowering coronary artery calcification while proximity to poorly maintained parks in deprived neighborhoods predicts higher risk, likely reflecting safety, utilization, and pollution gradients. Urban agriculture and community gardens illustrate how green spaces can also improve the food environment by enhancing access to fresh produce [64], reducing reliance on ultra-processed foods, and bolstering dietary quality. The combined influence of greenery, pollution buffering, walkability, and food access underscores the need for integrated planning strategies that create safe, high-quality, and nutritionally supportive green spaces capable of reducing cumulative environmental CVD burdens.

## The Social Environment

### Socioeconomic Status

Socioeconomic status (SES), including education, occupation, income, and wealth, strongly shapes exposure and vulnerability to environmental pollution. Lower-SES populations are more likely to live near highways, industrial facilities, and waste sites, resulting in higher chronic exposure to PM₂.₅, nitrogen oxides, and ozone [67]. Poor housing quality and limited air conditioning increase indoor pollutant infiltration, while socioeconomic adversity heightens biological susceptibility through chronic stress, nutritional deprivation, and reduced access to preventive care, amplifying inflammation and endothelial dysfunction [12, 58, 64]. Race and ethnicity intersect with SES [31, 68] through structural racism embedded in housing and zoning policies [14, 31, 68, 69]. Historical redlining and discriminatory land-use practices have concentrated pollution sources in communities of color, making race a dominant predictor of exposure independent of income. Pollution interacts with racism-related stress to accelerate vascular aging and reinforce cardiometabolic inequities.

### Family Structure and Psychosocial Stressors

Family structure, psychosocial stress, and social support jointly shape exposure to and vulnerability from environmental pollution across the life course. Single-parent households, overcrowding, and multigenerational caregiving are more prevalent in socioeconomically disadvantaged neighborhoods characterized by elevated ambient and indoor pollution [67, 68]. These contexts often coincide with substandard housing, proximity to traffic corridors, and limited residential mobility, increasing cumulative exposure. Early-life adversity and household instability may further sensitize neuroendocrine, immune, and metabolic pathways, rendering children and adolescents more susceptible to pollution-related cardiometabolic programming with persistent adult consequences [15, 46].

Psychosocial stressors, including job strain, neighborhood disorder, crime, noise, discrimination, and financial insecurity, operate synergistically with pollution through convergent biological mechanisms [70]. Chronic stress activates the sympathetic nervous system and hypothalamic–pituitary–adrenal axis, promoting cortisol dysregulation, oxidative stress, and systemic inflammation, pathways also triggered by air pollution [15, 71]. Population studies demonstrate interactive effects between neighborhood deprivation, adverse childhood experiences, and particulate matter on diabetes, stroke, and obesity, supporting a cumulative stress framework in which psychosocial adversity amplifies pollution-related CVD harm [10, 15, 71].

### The Policy Environment

Federal regulations, especially the Clean Air Act and subsequent Environmental Protection Agency (EPA) standards, have significantly reduced ambient PM and improved population health [72]. Regions with stronger regulatory enforcement consistently show lower environment-related mortality. Yet CVD risk persists at pollution levels below current NAAQS, and millions remain exposed above recommended thresholds. Updating the standards based on emerging epidemiologic and clinical evidence, and ensuring strict enforcement, remains essential to further reduce CVD burden [73]. Although the EPA strengthened NAAQS for fine PM in 2024, these evidence-based standards were later reversed during the second Trump administration [74]. The agency’s mission and capacity were significantly weakened through staffing reductions, closure of the Office of Research and Development, and a shift toward deregulation and energy production over environmental protection [25]. Similar political and structural barriers limit evidence-based air quality policies in many countries, despite clear scientific consensus on the benefits of emission reduction.

The global consequences of inaction are substantial. In India, long-term air pollution exposure contributed an estimated 1.8 million deaths in 2019, 18% of total mortality, relative to World Health Organization guideline levels [75]. Major sources include fossil fuel combustion, coal, and transportation. Economic losses are serious: air pollution cost India an estimated US$95 billion in 2019, or 3% of GDP, due to premature mortality, reduced productivity, and increased healthcare use [76]. Although multiple initiatives aim to improve air quality, major challenges remain in sustaining long-term pollution control.

Public awareness of air pollution’s health and economic effects remains limited, particularly in marginalized communities. Risk-communication systems are underdeveloped, and many clinicians lack training on environmental exposures and their CVD implications [77]. Environmental stressors remain insufficiently integrated into global health education and CVD risk assessment tools [78], despite escalating burden from pollution and climate change.

Strengthening public health infrastructure is critical. Priorities include expanding environmental health education, improving community engagement, promoting renewable energy and sustainable transport, increasing access to heart-healthy foods, and supporting local food systems that reinforce nutrition and economic resilience. Health impact assessments should be incorporated into zoning and infrastructure decisions to prevent additional pollution sources in overburdened communities [79]. Ultimately, effective policy requires a strong evidence base and sustained political will. Recent U.S. policy reversals, including weakened environmental protections and retreat from international cooperation [80] threaten progress. Delays will increase future health, economic, and environmental costs and make solutions more difficult to achieve.

### Lifecourse Windows of Susceptibility

A growing body of evidence indicates that CVD vulnerability to air pollution is not uniform across the lifespan but instead reflects critical windows of susceptibility during which exposures exert disproportionate and lasting effects [15, 46]. Prenatal exposure to PM₂.₅ can disrupt fetal development through oxidative stress, inflammation, and epigenetic reprogramming, thereby predisposing offspring to lifelong cardiometabolic dysfunction [15]. During infancy and childhood [46], the heightened plasticity of the immune, pulmonary, and CVD systems amplifies sensitivity to ambient pollutants, shaping long-term trajectories of endothelial function, autonomic balance, and metabolic regulation. Adolescence represents a transitional phase [15] in which environmental exposures interact with behavioral, hormonal, and psychosocial changes, potentially accelerating subclinical vascular injury. In older adulthood [46], age-related declines in physiological reserve magnify pollutant-induced CVD stress, consistent with a “harvesting effect” that increases vulnerability to acute events [52].

Across these stages, cumulative and repeated exposures contribute to progressive atherosclerosis, left ventricular remodeling, and worsening cardiometabolic risk, thereby amplifying susceptibility to both chronic disease progression and acute pollution-related triggers [15, 40, 52]. Importantly, these biological processes are embedded within broader social and built environments, where socioeconomic disadvantage, neighborhood stressors, and limited environmental control further intensify exposure burdens. Adopting a lifecourse framework is therefore essential for identifying sensitive periods, elucidating mechanisms of cumulative risk, and guiding early, multilevel interventions to mitigate lifelong CVD disease burden.

### Omics

Omics methods quantify cellular molecules and biological processes, including gene expression, metabolic activity, and protein levels. By capturing these molecular signatures, omics approaches enable the development of detailed exposure profiles that reveal pathways through which environmental exposures influence CVD risk across the life course. When integrated within the Public Health Exposome (PHE) framework, alongside data on the physical, social, and biological environments, these techniques can enhance risk assessment of environmental exposure [81, 82]. Emerging multi-omics studies [15, 16] show that air pollution, particularly PM₂.₅, induces coordinated epigenomic, proteomic, and metabolomic disruptions involving inflammation, oxidative stress, endothelial dysfunction, and lipid metabolism [15, 83]. These alterations converge on pathways promoting CVD [15]. Omics also reveal heterogeneity in susceptibility shaped by socioeconomic and built environments [15, 16]. Incorporating lifecourse perspectives into multi-omics research [15] can enhance early risk stratification, improve identification of populations at heightened risk, and support the development of targeted prevention strategies within epidemiologic practice.

Multi-omics studies demonstrate that air pollution, particularly PM₂.₅ and traffic-related emissions, induces coordinated disruptions across epigenomic, transcriptomic, proteomic, and metabolomic domains [15, 16, 83, 84]. Exposure has been linked to differential DNA methylation in genes regulating inflammation, oxidative stress, endothelial function, and DNA repair, driving transcriptional shifts that amplify pro-inflammatory and pro-atherogenic signaling [15, 84].

Proteomic profiles indicate elevated cytokine activity, acute-phase responses, and increased coagulation factors, consistent with systemic inflammation, endothelial injury, and thrombogenic potential [16]. Metabolomic profiles further reveal disruptions in lipid metabolism [15], mitochondrial function, and amino acid processing [83]. When examined jointly, these molecular layers converge on pathways implicated in vascular dysfunction and cardiometabolic disease and highlight variation in susceptibility shaped by socioeconomic and built environments [15, 16, 83, 84]. Incorporating a lifecourse perspective emphasizes how early-life exposures leave lasting molecular imprints, supporting the development of precision environmental cardiology approaches that enhance risk stratification and inform targeted prevention efforts.

### Methodological Advances: Deep Learning and Other AI

Rapid advances in deep learning and other artificial intelligence (AI) techniques are creating new opportunities to strengthen and integrate the global evidence base linking environmental risk factors, such as air and water pollution, noise, and heat stress, to CVD. However, analyzing the exposome and its relationship to CVD is exceptionally complex, requiring integration of high-dimensional, time-varying, and spatially resolved data that capture the totality of lifetime environmental exposures rather than isolated factors [17, 85–87]. Traditional epidemiological models, which often assume linear relationships, are not well suited to address the strong correlations, nonlinear relationships, and mixture effects inherent in exposomic data, particularly given evidence that chemical pollution such as PM₂.₅ can trigger systemic inflammation and oxidative stress, substantially increasing CVD morbidity. Integrating omics-based measures of the internal exposome with external environmental exposures poses additional challenges, including the alignment of temporal and spatial scales, the high dimensionality of molecular data, and the complexity of modeling joint associations with CVD risk. Persistent research gaps further hinder the operationalization of the PHE, as summarized in Table 2. These gaps include continued reliance on single-pollutant approaches, limited integration of built and social environments, insufficient characterization of molecular and endo-exposome pathways, inadequate life-course modeling, underutilization of flexible analytic methods, and challenges in translating exposomic evidence into effective policy.

**Table 2:** Summary of Key Research Gaps in Air Pollution and the Public Health Exposome

Domain	Persisting Gaps	Needed Advances
Environmental Mixtures	Focus on single pollutants; limited synergy models	Multi-exposure modeling, mixture toxicology
Contextual Factors	Limited integration of built/social environment	Multilevel exposure mapping; structural determinants
Molecular Integration	Incomplete endo-exposome signatures	Multi-omics, causal mediation
Lifecourse	Insufficient timing models	Window-specific susceptibility, cumulative burden
Methods	Linear models inadequate	ML/AI, graph analytics, high-resolution sensing
Policy Translation	Evidence–policy gap	Targeted public policy interventions

To address these challenges, researchers are increasingly applying advanced machine-learning and deep-learning methodologies [88], including random forests [89], gradient-boosted decision trees, neural networks, and ensemble algorithms such as XGBoost, to improve exposure assessment and prediction of CVD events by detecting nonlinear patterns and synergistic pollutants effects [90]. Graph-theoretical approaches further characterize correlated pollutant clusters and exposure networks, while Exposome-Wide Association Studies (ExWAS) and federated learning expand the capacity to examine diverse exposures across decentralized datasets [91]. Concurrently, wearable sensors, personal air monitors, GPS-enabled technologies, and machine vision technologies generate real-time, individualized, and street-level exposure data to support fine-grained spatiotemporal analytics [92]. Collectively, these innovations are advancing an integrated, systems-oriented exposome science capable of quantifying cumulative environmental stressors, integrating internal and external exposures, and identifying populations at heightened CVD risk.

## Conclusion

The transition to a PHE framework represents an essential evolution for contemporary CVD epidemiology. Traditional exposure–outcome approaches inadequately capture the complex intersection of the environmental, social, and structural determinants that shape CVD vulnerability in real-world settings. Individuals are not exposed to fine particulate matter in isolation; exposures co-occur with extreme heat, neighborhood deprivation, psychosocial stress, and the enduring effects of structural inequity. Emerging evidence indicates that these co-existing stressors act synergistically and produce spatially patterned risks across the life course.

By organizing natural, built, social, and policy environments into a cohesive systems structure, the PHE provides a framework to integrate complex mixtures, multilevel contextual influences, and biological response pathways. It underscores that CVD risk is shaped as much by structural and policy determinants as by biological susceptibility. Advances in high-resolution spatial modeling, satellite-based monitoring, machine learning, and causal inference approaches can further enhance the precision of risk characterization and the prediction of environmental stressors and CVD. The integration of exposomic science with robust, evidence-based policies offers a promising path toward reducing pollution-attributable CVD and improving health outcome for all.

## Key References

- Naghavi M, Kyu HH, Bhoomadevi A, Aalipour MA, Aalruz H, Ababneh HS, et al. Global burden of 292 causes of death in 204 countries and territories and 660 subnational locations, 1990–2023. Lancet. 2025.
  - ○ Findings from this comprehensive global study indicate that cardiovascular disease (CVD) remains the leading cause of death worldwide, accounting for nearly 50% of all mortality associated with non-communicable diseases.
- Münzel T, Lüscher T, Kramer CM, Churchwell K, Mbakwem A, Rajagopalan S. Environmental stressors and cardiovascular health: acting locally for global impact in a changing world: A joint statement of the ESC, ACC, AHA, and WHF. JACC. 2026.
  - ○ Findings from this landmark joint society statement establish air pollution and related environmental stressors as urgent, preventable determinants of CVD that require immediate integration into clinical practice and policy.
- Bakkensen LA, Ma L, Muehlenbachs L, Benitez L. Cumulative impacts in environmental justice: Insights from economics and policy. Reg Sci Urban Econ. 2024;107:103993.
  - ○ Findings from this study establish a framework for the cumulative impacts of environmental stressors, emphasizing that synergistic effects of pollution and social adversity are not captured by single-pollutant models.
- Liu M, Meijer P, Lam TM, Timmermans EJ, Grobbee DE, Beulens JWJ, et al. The built environment and cardiovascular disease: an umbrella review and meta-meta-analysis. Eur J Prev Cardiol. 2023;30(16):1801–27.
  - ○ Findings from this meta-meta-analysis indicate high-level evidence for consistent relationships between built environment features, such as walkability and transit access, and cardiovascular health outcomes.
- Noorali AA, Hussain Merchant AA, Afzal N, Sen R, Junaid V, Khoja A, et al. Built Environment and Cardiovascular Diseases – Insights from a Global Review. Curr Atheroscler Rep. 2025;27(1):36.
  - ○ Findings from this global review indicate that urban design and transportation infrastructure simultaneously structure pollution levels and opportunities for physical activity, directly influencing CVD prevalence.
- Khraishah H, Chen Z, Rajagopalan S. Understanding the cardiovascular and metabolic health effects of air pollution in the context of cumulative exposomic impacts. Circ Res. 2024;134(9):1083–97.
  - ○Findings from this study demonstrate how cumulative exposomic impacts across the lifecourse program cardiometabolic risk, specifically highlighting that early-life exposures leave lasting molecular imprints on vascular health.
- Perry AS, Zhang K, Murthy VL, Choi B, Zhao S, Gajjar P, et al. Proteomics, human environmental exposure, and cardiometabolic risk. Circ Res. 2024;135(1):138–54.
  - ○ Findings from this multi-omics research demonstrate that proteomic "molecular signatures" can accurately quantify an individual's internal biological response to external environmental stressors for precision risk stratification.
- Lane HM, Morello-Frosch R, Marshall JD, Apte JS. Historical Redlining Is Associated with Present-Day Air Pollution Disparities in U.S. Cities. Environ Sci Technol Lett. 2022;9(4):345–50.
  - ○ Findings from this study indicate that historical discriminatory housing and zoning policies, such as redlining, are direct predictors of present-day air pollution disparities and cardiovascular inequities.
- Kim K, Joyce BT, Zheng Y, Nannini DR, Wang J, Gordon-Larsen P, et al. Associations of urban blue and green spaces with coronary artery calcification in black individuals and disadvantaged neighborhoods. Circulation. 2024;150(3):203–14.
  - ○ Findings from this CARDIA cohort analysis reveal that while greenness generally lowers calcification, proximity to poorly maintained parks in deprived neighborhoods predicts higher cardiovascular risk due to safety and pollution gradients.
- Kershaw KN, Magnani JW, Diez Roux AV, et al. Neighborhoods and Cardiovascular Health: A Scientific Statement From the American Heart Association. Circ Cardiovasc Qual Outcomes. 2024;17(1):e000124.
  - ○ Findings from this scientific statement establish neighborhood-level social and physical structures as primary determinants of cardiovascular inequities, calling for screening tools to include environmental risk.
- Aguilar-Gomez S, Cardenas JC, Salas Diaz R. Environmental justice beyond race: Skin tone and exposure to air pollution. Proc Natl Acad Sci. 2025;122(10):e2407064122.
  - ○ Findings from this innovative research indicate that skin tone is a significant predictor of pollution exposure independent of income, revealing complex layers of structural racism within environmental health.
- Makram OM, Nwana N, Pan A, Nicolas JC, Gullapelli R, Bose B, et al. Interplay Between Residential Nature Exposure and Walkability and Their Association with Cardiovascular Health. JACC Adv. 2025;4(1):101457.
  - ○ Findings from this study indicate a significant interplay between nature exposure and walkability, where their combined influence buffers pollution-related autonomic stress and promotes healthy metabolic regulation.

## Data Availability

No datasets were generated or analysed during the current study.

## Declarations

Human and Animal Rights and Informed Consent This article does not contain any studies with human or animal subjects performed by any of the authors.

## References

[1] Naghavi M, Kyu HH, Bhoomadevi A, Aalipour MA, Aalruz H, Ababneh HS et al. Global burden of 292 causes of death in 204 countries and territories and 660 subnational locations. 2025;1990–2023. 10.1016/S0140-6736(25)01917-8 PMC12535838 41092928

[2] Roth GA, Mensah GA, Johnson CO, Addolorato G, Ammirati E, Baddour LM, et al. Global burden of cardiovascular diseases and risk factors, 1990–2019: update from the GBD 2019 study. J Am Coll Cardiol. 2020;76(25):2982–3021. 33309175 10.1016/j.jacc.2020.11.010 PMC7755038

[3] World Health Organization. WHO global air quality guidelines: particulate matter (PM2. 5 and PM10), ozone, nitrogen dioxide, sulfur dioxide and carbon monoxide. World Health Organization. 2021. Sep 7. 34662007

[4] Juarez PD, Hood DB, Song M-A, Ramesh A. Use of an Exposome Approach to Understand the Effects of Exposures From the Natural, Built, and Social Environments on Cardio-Vascular Disease Onset, Progression, and Outcomes. Front Public Health. 2020; 8–2020. 10.3389/fpubh.2020.00379 PMC7437454 32903514

[5] Juarez PD, Matthews-Juarez P, Hood DB, Im W, Levine RS, Kilbourne BJ, et al. The public health exposome: a population-based, exposure science approach to health disparities research. Int J Environ Res Public Health. 2014;11(12):12866–95. 25514145 10.3390/ijerph111212866 PMC4276651

[6] Wild CP. The exposome: from concept to utility. Int J Epidemiol. 2012;41(1):24–32. 22296988 10.1093/ije/dyr236

[7] Münzel T, Lüscher T, Kramer CM, Churchwell K, Mbakwem A, Rajagopalan S. Environmental stressors and cardiovascular health: acting locally for global impact in a changing world: A statement of the European Society of Cardiology, the American College of Cardiology, the American Heart Association, and the World Heart Federation. JACC. 2026. 10.5334/gh.1514 PMC12829457 41586406

[8] Wild CP. Complementing the genome with an exposome: the outstanding challenge of environmental exposure measurement in molecular epidemiology. Cancer Epidemiol Biomarkers Prev. 2005;14(8):1847–50. 16103423 10.1158/1055-9965.EPI-05-0456

[9] Daiber A, Lelieveld J, Steven S, Oelze M, Kröller-Schön S, Sørensen M, et al. The “exposome” concept - how environmental risk factors influence cardiovascular health. Acta Biochim Pol. 2019;66(3):269–83. 31509369 10.18388/abp.2019_2853

[10] Bakkensen LA, Ma L, Muehlenbachs L, Benitez L. Cumulative impacts in environmental justice: insights from economics and policy. Reg Sci Urban Econ. 2024;107:103993.

[11] Liu M, Meijer P, Lam TM, Timmermans EJ, Grobbee DE, Beulens JWJ, et al. The built environment and cardiovascular disease: an umbrella review and meta-meta-analysis. Eur J Prev Cardiol. 2023;30(16):1801–27. 37486178 10.1093/eurjpc/zwad241

[12] Noorali AA, Hussain Merchant AA, Afzal N, Sen R, Junaid V, Khoja A, et al. Built environment and cardiovascular diseases – insights from a global review. Curr Atheroscler Rep. 2025;27(1):36. 40042532 10.1007/s11883-025-01282-2

[13] Garcia A, Santa-Helena E, De Falco A, de Paula Ribeiro J, Gioda A, Gioda CR. Toxicological effects of fine particulate matter (PM2. 5): health risks and associated systemic injuries—systematic review. Water Air Soil Pollut. 2023;234(6):346. 37250231 10.1007/s11270-023-06278-9 PMC10208206

[14] Lane HM, Morello-Frosch R, Marshall JD, Apte JS. Historical redlining is associated with present-day air pollution disparities in U.S. cities. Environ Sci Technol Lett. 2022;9(4):345–50. 35434171 10.1021/acs.estlett.1c01012 PMC9009174

[15] Khraishah H, Chen Z, Rajagopalan S. Understanding the cardiovascular and metabolic health effects of air pollution in the context of cumulative exposomic impacts. Circ Res. 2024;134(9):1083–97. 38662860 10.1161/CIRCRESAHA.124.323673 PMC11253082

[16] Perry AS, Zhang K, Murthy VL, Choi B, Zhao S, Gajjar P, et al. Proteomics, human environmental exposure, and cardiometabolic risk. Circ Res. 2024;135(1):138–54. 38662804 10.1161/CIRCRESAHA.124.324559 PMC11189739

[17] Argyri KD, Gallos IK, Amditis A, Dionysiou DD. Exposomics and cardiovascular diseases: a scoping review of machine learning approaches. medRxiv. 2025:2024.07.19.24310695.

[18] Hamanaka RB, Mutlu GM. Particulate matter air pollution: effects on the respiratory system. J Clin Invest. 2025. 10.1172/JCI194312. 40892514 10.1172/JCI194312 PMC12404767

[19] Wibowo A. The Growing Burden of Climate Change and Air Pollution on COPD Morbidity and Mortality.

[20] Hamanaka RB, Mutlu GM. Particulate matter air pollution: effects on the cardiovascular system. Front Endocrinol (Lausanne). 2018;9:680. 30505291 10.3389/fendo.2018.00680 PMC6250783

[21] Krittanawong C, Qadeer YK, Hayes RB, Wang Z, Thurston GD, Virani S, et al. PM2. 5 and cardiovascular diseases: state-of-the-art review. International Journal of Cardiology Cardiovascular Risk and Prevention. 2023;19:200217. 37869561 10.1016/j.ijcrp.2023.200217 PMC10585625

[22] Guan L, Geng X, Stone C, Cosky EE, Ji Y, Du H, et al. PM2. 5 exposure induces systemic inflammation and oxidative stress in an intracranial atherosclerosis rat model. Environ Toxicol. 2019;34(4):530–8. 30672636 10.1002/tox.22707

[23] Thangavel P, Park D, Lee Y-C. Recent insights into particulate matter (PM2. 5)-mediated toxicity in humans: an overview. Int J Environ Res Public Health. 2022;19(12):7511. 35742761 10.3390/ijerph19127511 PMC9223652

[24] Agency USEP. EPA Finalizes Stronger Standards for Harmful Soot Pollution, Significantly Increasing Health and Clean Air Protections for Families, Workers, and Communities Washington, DC: U.S. Environmental Protection Agency; 2024 [updated February 7, 2024. Available from: https://www.epa.gov/newsreleases/epa-finalizes-stronger-standards-harmful-soot-pollution-significantly-increasing.

[25] Brown C, Looi M-K. Trump repeals EPA climate change regulations in move scientists say is a “rejection of the laws of physics.” BMJ. 2026. 10.1136/bmj.s324. 41702650 10.1136/bmj.s324

[26] Rizzo LV, Rizzo MCF. Wildfire smoke and health impacts: a narrative review. J Pediatr (Rio J). 2025;101:S56-64. 39681318 10.1016/j.jped.2024.11.006 PMC11962561

[27] Miller MR, Landrigan PJ, Arora M, Newby DE, Münzel T, Kovacic JC. Environmentally not so friendly: global warming, air pollution, and wildfires: JACC focus seminar, part 1. J Am Coll Cardiol. 2024;83(23):2291–307. 38839204 10.1016/j.jacc.2024.03.424 PMC11908388

[28] Issakhov A, Abylkassymova A. The impact of humidity and road surface temperature varying by daytime on the dispersion of air pollutants. Int J Environ Sci Technol. 2025;22(16):16351–84.

[29] Tsekeri E, Lilli A, Lazaridis M, Kolokotsa D. Air pollution in the urban built environment: A comprehensive evaluation. Atmospheric Pollution Res. 2025:102797.

[30] Khan RN, Saporito AF, Zenon J, Goodman L, Zelikoff JT. Traffic-related air pollution in marginalized neighborhoods: a community perspective. Inhal Toxicol. 2024;36(5):343–54. 38618680 10.1080/08958378.2024.2331259

[31] Aguilar-Gomez S, Cardenas JC, Salas Diaz R. Environmental justice beyond race: skin tone and exposure to air pollution. Proc Natl Acad Sci USA. 2025;122(10):e2407064122. 40035760 10.1073/pnas.2407064122 PMC11912393

[32] Bevan GH, Al-Kindi SG, Brook RD, Münzel T, Rajagopalan S. Ambient air pollution and atherosclerosis. Arterioscler Thromb Vasc Biol. 2021;41(2):628–37. 33327745 10.1161/ATVBAHA.120.315219

[33] Brook RD, Motairek I, Rajagopalan S, Al-Kindi S. Excess global blood pressure associated with fine particulate matter air pollution levels exceeding World Health Organization guidelines. J Am Heart Assoc. 2023;12(8):e029206. 37042288 10.1161/JAHA.122.029206 PMC10227265

[34] de Bont J, Jaganathan S, Dahlquist M, Persson Å, Stafoggia M, Ljungman P. Ambient air pollution and cardiovascular diseases: an umbrella review of systematic reviews and meta-analyses. J Intern Med. 2022;291(6):779–800. 35138681 10.1111/joim.13467 PMC9310863

[35] Mayntz SP, Rosenbech KE, Mohamed RA, Lindholt JS, Diederichsen ACP, Frohn LM, et al. Impact of air pollution and noise exposure on cardiovascular disease incidence and mortality: a systematic review. Heliyon. 2024;10(21):e39844. 39524794 10.1016/j.heliyon.2024.e39844 PMC11550137

[36] Rajagopalan S, Brook RD, Salerno PRVO, Bourges-Sevenier B, Landrigan P, Nieuwenhuijsen MJ, et al. Air pollution exposure and cardiometabolic risk. Lancet Diabetes Endocrinol. 2024;12(3):196–208. 38310921 10.1016/S2213-8587(23)00361-3 PMC11264310

[37] Rajagopalan S, Landrigan PJ. Pollution and the heart. N Engl J Med. 2021;385(20):1881–92. 34758254 10.1056/NEJMra2030281

[38] Montone RA, Rinaldi R, Bonanni A, Severino A, Pedicino D, Crea F, et al. Impact of air pollution on ischemic heart disease: Evidence, mechanisms, clinical perspectives. Atherosclerosis. 2023;366:22–31. 36696748 10.1016/j.atherosclerosis.2023.01.013

[39] Bevan GH, Al-Kindi SG, Brook R, Rajagopalan S. Ambient air pollution and atherosclerosis: recent updates. Curr Atheroscler Rep. 2021;23(10):63. 34417890 10.1007/s11883-021-00958-9 PMC8379601

[40] Mohamed RA, Peronard Mayntz S, Møller J-JK, Mejldal A, Schultz Overgaard K, Rueskov Andersen T et al. Long-term ambient air pollution exposure and coronary calcium score in men: insights from the DANCAVAS study. Eur J Prev Cardiol. 2025. 10.1093/eurjpc/zwaf688 41140071

[41] Zhao D, Yao J, Li Y, Jacobs DR Jr, Gao T, Nie LH, et al. Urban imperviousness and carotid intima-medial thickness: Evidence from the Coronary Artery Risk Development in Young Adults (CARDIA) study. Sci Total Environ. 2025;978:179376. 40252492 10.1016/j.scitotenv.2025.179376 PMC12323400

[42] Wang K, Lei L, Li G, Lan Y, Wang W, Zhu J, et al. Association between Ambient Particulate Air Pollution and Soluble Biomarkers of Endothelial Function: A Meta-Analysis. Toxics. 2024;12(1):76. 38251031 10.3390/toxics12010076 PMC10819696

[43] Scheuermann BC, Parr SK, Schulze KM, Kunkel ON, Turpin VRG, Liang J, et al. Associations of cerebrovascular regulation and arterial stiffness with cerebral small vessel disease: a systematic review and meta-analysis. J Am Heart Assoc. 2023;12(23):e032616. 37930079 10.1161/JAHA.123.032616 PMC10727345

[44] Ding R, Ren X, Sun Q, Yang M, Wang Y, Sun Z, et al. Air pollution and stroke: an emerging challenge from cardio-cerebrovascular multimorbidity. J Am Heart Assoc. 2025;14(13):e041848. 40576036 10.1161/JAHA.124.041848 PMC12449924

[45] Ma Y, Hui Y, Tang L, Wang J, Xing M, Zheng L, et al. Ambient air pollution exposure in relation to cerebral small vessel disease in Chinese population: A cranial magnetic resonance imaging-based study. Eco-Environment Health. 2025;4(1):100129. 39925481 10.1016/j.eehl.2024.10.004 PMC11803214

[46] Hahad O, Frenis K, Kuntic M, Daiber A, Münzel T. Accelerated Aging and Age-Related Diseases (CVD and Neurological) Due to Air Pollution and Traffic Noise Exposure. Int J Mol Sci. 2021;22(5):2419. 33670865 10.3390/ijms22052419 PMC7957813

[47] Guo L-H, Zeeshan M, Huang G-F, Chen D-H, Xie M, Liu J, et al. Influence of air pollution exposures on cardiometabolic risk factors: a review. Current Environmental Health Reports. 2023;10(4):501–7. 38030873 10.1007/s40572-023-00423-6

[48] Nielsen K, Andersen Z, Cramer J, Amini H, Lim Y. Long-term exposure to air pollution and heart failure: a systematic review and meta-analyses. ISEE Conference Abstracts. 2020. 10.1289/isee.2020.virtual.O-PK-2081.

[49] Zhang L, Liu Z, Zhou X, Zeng J, Wu M, Jiang M. Long-term impact of air pollution on heart failure readmission in unstable angina patients. Sci Rep. 2024;14(1):22132. 39333793 10.1038/s41598-024-73495-5 PMC11436851

[50] Dabass A, Talbott EO, Rager JR, Marsh GM, Venkat A, Holguin F, et al. Systemic inflammatory markers associated with cardiovascular disease and acute and chronic exposure to fine particulate matter air pollution (PM2. 5) among US NHANES adults with metabolic syndrome. Environ Res. 2018;161:485–91. 29223110 10.1016/j.envres.2017.11.042

[51] Li G, Jia Y, Cui Y, Wu S, Ma T, Jiang Y, et al. Air Pollution and Cardiac Biomarkers in Heart Failure: A Scoping Review. Biomed Environ Sci. 2025;38(11):1430–43. 41368966 10.3967/bes2025.120

[52] Donzelli G, Sera F, Morales MA, Vozzi F, Roos T, Schaffert A, et al. A systematic review and meta-analysis of human population studies on the association between exposure to toxic environmental chemicals and left ventricular dysfunction (LVD). Environ Res. 2024;249:118429. 38354889 10.1016/j.envres.2024.118429

[53] Zhang Y, Shi J, Ma Y, Yu N, Zheng P, Chen Z, et al. Association between air pollution and lipid profiles. Toxics. 2023;11(11):894. 37999546 10.3390/toxics11110894 PMC10675150

[54] Jaganathan S, Jaacks LM, Magsumbol M, Walia GK, Sieber NL, Shivasankar R, et al. Association of long-term exposure to fine particulate matter and cardio-metabolic diseases in low- and middle-income countries: a systematic review. Int J Environ Res Public Health. 2019;16(14):2541. 31315297 10.3390/ijerph16142541 PMC6679147

[55] Li R, Cai M, Qian ZM, Wang X, Zhang Z, Wang C, et al. Ambient air pollution, lifestyle, and genetic predisposition associated with type 2 diabetes: findings from a national prospective cohort study. Sci Total Environ. 2022;849:157838. 35934032 10.1016/j.scitotenv.2022.157838

[56] Jiang Y, Huang J, Li G, Wang W, Wang K, Wang J, et al. Ozone pollution and hospital admissions for cardiovascular events. Eur Heart J. 2023;44(18):1622–32. 36893798 10.1093/eurheartj/ehad091

[57] Xia X, Meng X, Liu C, Guo Y, Li X, Niu Y, et al. Associations of long-term nitrogen dioxide exposure with a wide spectrum of diseases: a prospective cohort study of 0· 5 million Chinese adults. Lancet Public Health. 2024;9(12):e1047–58. 39643329 10.1016/S2468-2667(24)00264-0 PMC11626078

[58] Li J, Liu F, Liang F, Yang Y, Lu X, Gu D. Air pollution exposure and vascular endothelial function: a systematic review and meta-analysis. Environ Sci Pollut Res Int. 2023;30(11):28525–49. 36702984 10.1007/s11356-023-25156-9

[59] Rajagopalan S, Park B, Palanivel R, Vinayachandran V, Deiuliis JA, Gangwar RS, et al. Metabolic effects of air pollution exposure and reversibility. J Clin Investig. 2020;130(11):6034–40. 32780721 10.1172/JCI137315 PMC7598058

[60] Joyce BT, Yao J, Zheng Y, Gao T, Nannini D, Lin S, et al. Temperature and carotid intima-medial thickness: The coronary artery risk development in young adults (CARDIA) study. Sci Total Environ. 2024;954:176573. 39343405 10.1016/j.scitotenv.2024.176573 PMC12356224

[61] Klompmaker JO, Laden F, James P, Benjamin Sabath M, Wu X, Dominici F, et al. Long-term exposure to summer specific humidity and cardiovascular disease hospitalizations in the US Medicare population. Environ Int. 2023;179:108182. 37683506 10.1016/j.envint.2023.108182 PMC10545022

[62] Wu Y, Feng X, Li J, Li M, Wang Y, Lu W, et al. Exposure to high-temperature and high-humidity environments associated with cardiovascular mortality. Ecotoxicol Environ Saf. 2025;290:117746. 39823668 10.1016/j.ecoenv.2025.117746

[63] Makram OM, Nwana N, Pan A, Nicolas JC, Gullapelli R, Bose B, et al. Interplay Between Residential Nature Exposure and Walkability and Their Association with Cardiovascular Health. JACC: Adv. 2025;4(1):101457. 39801816 10.1016/j.jacadv.2024.101457 PMC11719309

[64] Liu M, Patel VR, Salas RN, Rice MB, Kazi DS, Zheng Z, et al. Neighborhood environmental burden and cardiovascular health in the US. JAMA Cardiol. 2024;9(2):153–63. 37955891 10.1001/jamacardio.2023.4680 PMC10644252

[65] Liang J, Deng S, Yang H, Zhu S, Zheng R. Spatiotemporal effects of urban micro-scale built environment on cardiovascular diseases. Sci Rep. 2025;15(1):17193. 40382476 10.1038/s41598-025-02603-w PMC12085694

[66] Kim K, Joyce BT, Zheng Y, Nannini DR, Wang J, Gordon-Larsen P, et al. Associations of urban blue and green spaces with coronary artery calcification in black individuals and disadvantaged neighborhoods. Circulation. 2024;150(3):203–14. 38934130 10.1161/CIRCULATIONAHA.123.067992 PMC11250927

[67] Tessum CW, Paolella DA, Chambliss SE, Apte JS, Hill JD, Marshall JD. PM2. 5 polluters disproportionately and systemically affect people of color in the United States. Science advances. 2021;7(18):eabf4491. 33910895 10.1126/sciadv.abf4491 PMC11426197

[68] Kershaw KN, Magnani JW, Diez Roux AV, Camacho-Rivera M, Jackson EA, Johnson AE, et al. Neighborhoods and cardiovascular health: a scientific statement from the American Heart Association. Circulation: Cardiovascular Quality and Outcomes. 2024;17(1):e000124. 38073532 10.1161/HCQ.0000000000000124

[69] Bailey ZD, Krieger N, Agénor M, Graves J, Linos N, Bassett MT. Structural racism and health inequities in the USA: evidence and interventions. Lancet. 2017;389(10077):1453–63. 28402827 10.1016/S0140-6736(17)30569-X

[70] Hicken MT, Dvonch JT, Schulz AJ, Mentz G, Max P. Fine particulate matter air pollution and blood pressure: the modifying role of psychosocial stress. Environ Res. 2014;133:195–203. 24968081 10.1016/j.envres.2014.06.001 PMC4137402

[71] Xu H, Yang T, Guo B, Silang Y, Dai Y, Baima K, et al. Increased allostatic load associated with ambient air pollution acting as a stressor: Cross-sectional evidence from the China multi-ethnic cohort study. Sci Total Environ. 2022;831:155658. 35523330 10.1016/j.scitotenv.2022.155658

[72] Agency UEP. The benefits and costs of the Clean Air Act from 1990 to 2020. Washington, DC: US Environmental Protection Agency; 2011.

[73] Kaufman JD, Elkind MSV, Bhatnagar A, Koehler K, Balmes JR, Sidney S, et al. Guidance to reduce the cardiovascular burden of ambient air pollutants: a policy statement from the American Heart Association. Circulation. 2020;142(23):e432-47. 33147996 10.1161/CIR.0000000000000930

[74] Witze A. US repeals key ‘endangerment finding’that climate change is a public threat. Nature. 2026;650(8103):806–7. 41680522 10.1038/d41586-026-00455-6

[75] Jaganathan S, Stafoggia M, Rajiva A, Mandal S, Dixit S, De Bont J, et al. Estimating the effect of annual PM2· 5 exposure on mortality in India: a difference-in-differences approach. Lancet Planet Health. 2024;8(12):e987–96. 39674205 10.1016/S2542-5196(24)00248-1 PMC11790315

[76] Advisors D. Air Pollution and its Impact on Business: The Silent Pandemic. London, UK: Clean Air Fund; 2021.

[77] Hadley MB, Baumgartner J, Vedanthan R. Developing a clinical approach to air pollution and cardiovascular health. Circulation. 2018;137(7):725–42. 29440198 10.1161/CIRCULATIONAHA.117.030377 PMC5950725

[78] Jacobsen KH, Waggett CE, Berenbaum P, Bayles BR, Carlson GL, English R, et al. Planetary health learning objectives: foundational knowledge for global health education in an era of climate change. Lancet Planet Health. 2024;8(9):e706–13. 39243786 10.1016/S2542-5196(24)00167-0

[79] Corburn J. Concepts for studying urban environmental justice. Curr Environ Health Rep. 2017;4(1):61–7. 28101730 10.1007/s40572-017-0123-6

[80] Knox JH, Bodansky D, Rajamani L. The Trump administration steps back from international environmental cooperation. Am J Int Law. 2025;119(4):767–77.

[81] Riggs DW, Yeager RA, Bhatnagar A. Defining the human envirome. Circ Res. 2018;122(9):1259–75. 29700071 10.1161/CIRCRESAHA.117.311230 PMC6398443

[82] Canali S, Leonelli S. Reframing the environment in data-intensive health sciences. Stud Hist Philos Sci. 2022;93:203–14. 35576883 10.1016/j.shpsa.2022.04.006

[83] Scimeca M, Palumbo V, Giacobbi E, Servadei F, Casciardi S, Cornella E, et al. Impact of the environmental pollution on cardiovascular diseases: from epidemiological to molecular evidence. Heliyon. 2024. 10.1016/j.heliyon.2024.e38047. 39328571 10.1016/j.heliyon.2024.e38047 PMC11425171

[84] Riggs DW, Yeager RA, Bhatnagar A. Defining the human envirome: an omics approach for assessing the environmental risk of cardiovascular disease. Circ Res. 2018;122(9):1259–75. 29700071 10.1161/CIRCRESAHA.117.311230 PMC6398443

[85] Isola S, Murdaca G, Brunetto S, Zumbo E, Tonacci A, Gangemi S, editors. The use of artificial intelligence to analyze the exposome in the development of chronic diseases: a review of the current literature. Informatics: MDPI; 2024.

[86] Muenzel T, Sørensen M, Hahad O, Nieuwenhuijsen M, Daiber A. The contribution of the exposome to the burden of cardiovascular disease. Nat Rev Cardiol. 2023;20(10):651–69. 37165157 10.1038/s41569-023-00873-3

[87] Ibrahim R, Pham HN, Nasir K, Hahad O, Sabharwal A, Al-Kindi S. Big data, big insights: leveraging data analytics to unravel cardiovascular exposome complexities. Methodist Debakey Cardiovasc J. 2024;20(5):111. 39525379 10.14797/mdcvj.1467 PMC11546329

[88] Maitre L, Guimbaud J-B, Warembourg C, Güil-Oumrait N, Petrone PM, Chadeau-Hyam M, et al. State-of-the-art methods for exposure-health studies: results from the exposome data challenge event. Environ Int. 2022;168:107422. 36058017 10.1016/j.envint.2022.107422

[89] Monaco A, Lacalamita A, Amoroso N, D’Orta A, Del Buono A, di Tuoro F, et al. Random forests highlight the combined effect of environmental heavy metals exposure and genetic damages for cardiovascular diseases. Appl Sci. 2021;11(18):8405.

[90] Atehortúa A, Gkontra P, Camacho M, Diaz O, Bulgheroni M, Simonetti V, et al. Cardiometabolic risk estimation using exposome data and machine learning. Int J Med Inform. 2023;179:105209. 37729839 10.1016/j.ijmedinf.2023.105209

[91] Chung MK, House JS, Akhtari FS, Makris KC, Langston MA, Islam KT, et al. Decoding the exposome: data science methodologies and implications in exposome-wide association studies (ExWASs). Exposome. 2024;4(1):osae001. 38344436 10.1093/exposome/osae001 PMC10857773

[92] Johnson T, Kanjo E, Woodward K. Digitalexposome: quantifying impact of urban environment on wellbeing using sensor fusion and deep learning. Computational urban science. 2023;3(1):14. 36970599 10.1007/s43762-023-00088-9 PMC10025809

